# Step Length Estimation Using Handheld Inertial Sensors

**DOI:** 10.3390/s120708507

**Published:** 2012-06-25

**Authors:** Valérie Renaudin, Melania Susi, Gérard Lachapelle

**Affiliations:** PLAN Group, Schulich School of Engineering, The University of Calgary, 2500 University Drive NW, Calgary, AB T2N 1N4, Canada; E-Mails: msusi@ucalgary.ca (M.S.); gerard.lachapelle@ucalgary.ca (G.L.)

**Keywords:** biomechanics, dead reckoning, pedestrian navigation, step length, handheld devices, IMU

## Abstract

In this paper a novel step length model using a handheld Micro Electrical Mechanical System (MEMS) is presented. It combines the user's step frequency and height with a set of three parameters for estimating step length. The model has been developed and trained using 12 different subjects: six men and six women. For reliable estimation of the step frequency with a handheld device, the frequency content of the handheld sensor's signal is extracted by applying the Short Time Fourier Transform (STFT) independently from the step detection process. The relationship between step and hand frequencies is analyzed for different hand's motions and sensor carrying modes. For this purpose, the frequency content of synchronized signals collected with two sensors placed in the hand and on the foot of a pedestrian has been extracted. Performance of the proposed step length model is assessed with several field tests involving 10 test subjects different from the above 12. The percentages of error over the travelled distance using universal parameters and a set of parameters calibrated for each subject are compared. The fitted solutions show an error between 2.5 and 5% of the travelled distance, which is comparable with that achieved by models proposed in the literature for body fixed sensors only.

## 1. Introduction

Recent progress in Micro Electro Mechanical Systems (MEMS) technology is stimulating their use in different domains including pedestrian navigation, location based services (LBS), safety and healthcare services. Because they are already embedded in many electronic devices, and particularly in smart phones, it is now possible to use these low cost sensors for developing new services, pedestrian navigation being one of the most attractive for the consumer grade market. Indeed MEMS accelerometers and gyroscopes provide autonomous solutions for tracking pedestrians in different types of environments, thereby overcoming the limitations of Global Navigation Satellite Systems (GNSS) in challenging contexts, such as indoors or urban canyons, where satellite signals are blocked or strongly attenuated by man-made infrastructures. Beyond using pedestrian navigation for commercial applications, the ability to continuously track people anywhere can answer safety issues such as monitoring vulnerable patients. This is, for example, the case for subject affected by a cognitive function deficit. Helping elderly people suffering from dementia or Alzheimer's is a good illustration. Indeed the main effects of these pathologies are memory loss and attention deficit inducing difficult orientation and wandering. These kinds of patients require continuous tracking and monitoring to provide prompt assistance in case of necessity or to alert third parties when they wander beyond a specific radius considered as a “safe” zone. However, MEMS sensors cannot provide an accurate self-contained system mainly due to the errors inherent to their low-cost nature, namely drift and biases affecting their signals [1]. Frequent external sources of information, for example GNSS updates, are needed for mitigating the sensors errors.

When GNSS aiding is not available alternative approaches must be adopted. For pedestrian navigation, the characteristics of human gait can be exploited in Pedestrian Dead Reckoning (PDR) algorithms instead of double integrating the inertial data, which is implemented in the strap-down method. Indeed the latter approach is not suitable for low-cost sensor based applications, since the double integration increases the noise component proportionally to the operational time even if the pedestrian is not moving. Instead in a PDR approach, the estimation of the current pedestrian's position results from the displacement of the user, *i.e.*, linear walked distance and walking direction, since the last known position estimate. This recursive process is related to the effective motion of the user.

The computation of the user's linear displacement generally consists of two parts: first detecting the user's steps and second evaluating their length. Estimating a pedestrian's step length is a challenging task that can be performed following different approaches, which strongly depend on the sensor's location. The majority of existing algorithms assumes that the sensor is rigidly attached to the user's body either on the foot, close to the Centre Of Mass (COM), e.g., along the backbone, or distributed on the leg [2–6]. These locations are particularly suitable for navigation purposes since the inertial force experienced by the sensor is directly linked to the gait cycle. Using body fixed sensors, two main categories of step length models can be identified in the literature: biomechanical and parametric models. In general biomechanical models assume that the sensor is located on the user's COM and model the user's leg as an inverted pendulum [5,6]. A simple geometric relationship between the COM's vertical displacement and the step length is then applied. Models based on other geometric considerations are also proposed in [7,8]. Parametric models use the step frequency and the accelerometers variance, either combined or independently, to estimate the step length [9,10]. Again the sensor is either mounted on the belt or on the foot but body fixed locations are not suitable for many applications. As explained, MEMS are often already embedded in unobtrusive portable devices, e.g., smart phones or personal digital assistants, which are usually carried in hands or kept in bags and therefore are “non-body fixed”. Published work on using non-body fixed sensors for pedestrian navigation is however often constraining the sensor's location to emplacements where the device is relatively stable while the user is walking. For example the device is carried in the user's trouser pocket [11] or constrained to specific locations like close to the ear while phoning or pointing toward the walking direction [12]. The reason is that in these scenarios, the IMU (Inertial Measurement Unit) signal patterns of the device are closer to the ones produced by body fixed sensors and subsequently similar approaches can be adopted.

When the sensor is handheld without any constraint, the situation becomes much more complex adding many new issues that require specific processing. For example, since the hand undergoes many motions which don't reflect the user's displacement, they have to be identified and classified as parasite in order to avoid wrong propagation of the user's position. In hand, not only is the sensor's orientation unknown *a priori* but also it can vary very quickly due to fast hand motions. In fact very few studies target the handheld sensor case and in general only the case of sensors held in the user's phoning or texting hand is considered [13]. Indeed in this context, the sensor is mainly experiencing the inertial force produced by the global motion of the user, which is similar to the body fixed case. Conversely, the cases of the sensor held in the swinging hand and when the sensor's placement changes while the user is moving are omitted. In [14], different sensor carrying modes are examined, including carrying the sensor in the swinging hand, but only traditional techniques, designed for body fixed sensors, are adopted. When the above techniques are applied to handheld smart phones, they produce lower performance than the ones obtained with body fixed sensors.

Facing the identified limitations of existing techniques in the context of autonomous indoor navigation based on smart phones, a dedicated and extensive analysis of the hand case has been performed herein. Its results are presented in this paper and lead to the development of a handheld based step length model. Algorithms are proposed for estimating the step length of pedestrians walking on a flat ground using handheld devices without constraining the sensor's carrying mode. The proposed step length model combines the user's step frequency and height. Step frequency evaluation is performed directly in the frequency domain and independently from the step detection process. In order to adapt the model to the handheld case, the relationship between step frequency and hand frequency is deeply investigated. Performance of the proposed model is assessed in the position domain by combining the step length model with a step detection algorithm presented in [15]. The assessment part, performed with 10 test subjects, shows that the handheld step length model achieves comparable performances as the ones obtained in the literature but with body fixed sensors only.

The structure of the paper is the following. In Section 2, the signal model is introduced and the signal preprocessing phase is illustrated. In Section 3, the analysis of human gait using handheld devices is described. Then, in Section 4, the proposed step length model is presented with a description of a novel technique used to extract the user's step frequency from the user's hand frequency. Section 5 deals with the assessment of the proposed algorithm with 10 test subjects. Finally, Section 6 draws conclusions.

## 2. Signal Model and Pre-Processing

In this paper step length estimation is performed using a six-degree of freedom (6DoF) IMU. It comprises a tri-axis gyroscope and accelerometer that sense angular rates and accelerations of the body frame. The sensor's digital output is modeled as a six-dimensional vector given by the sum of the response to the sensed inertial force and a noise term [12]:

(1) $$\text{s}\left[n\right]=\left[\begin{array}{l} {\text{s}}^{a}\left[n\right]\\ {\text{s}}^{\omega }\left[n\right] \end{array}\right]=\left[\begin{array}{l} \text{a}\left[n\right]\\ \text{ω}\left[n\right] \end{array}\right]+\left[\begin{array}{l} {\text{η}}^{a}\left[n\right]\\ {\text{η}}^{\omega }\left[n\right] \end{array}\right]$$

where:

- *n* ∈ ℕ, is the temporal index of the signal after sampling at frequency f_s_ = 1/Ts. For the experiments conducted in this paper f_s_ equals 100 Hz.
- ***s**^a^*[*n*] ∈ ℝ^3^, is the digital output of the tri-axis accelerometer composed by the acceleration vector ***a***[*n*] and the related noise vector ***η**^a^*[*n*].
- ***s**^ω^*[*n*] ∈ ℝ^3^, is the digital output of the tri-axis gyroscope composed by the angular rate vector and the related noise vector ***η**^ω^*[*n*].

Since the frequency content of the accelerations and angular rates induced by human gait are below 15 Hz [16], the components in Equation (1) are low pass filtered using a zero-lag 10th order Butterworth filter with a 15 Hz cut-off frequency. It is worth underlining that although fast hand motions produce frequencies larger than 15 Hz, these events are not of interest for pedestrian navigation because they are not related to the user's global locomotion and consequently can be removed. The filtered components are indicated with ***s̃**^a^* [*n*] and ***s̃**^ω^* [*n*].

When the sensor is not body fixed, the orientation of the handheld device is *a priori* unknown, which implies that the signal processing has to be immune to any change of the sensor's orientation. This is even more important with a handheld device as the rapid motions characteristic of the hand render the task of continuously estimating the device's orientation very challenging. Therefore, instead of working on the individual vector elements, the norm of the filtered components is considered:

(2) $${\tilde{\text{s}}}_{\text{rms}}\left[n\right]=\left[\begin{array}{l} {\tilde{s}}_{\text{rms}}^{a}\left[n\right]\\ {\tilde{s}}_{\text{rms}}^{\omega }\left[n\right] \end{array}\right]$$

where ${\tilde{s}}_{\text{rms}}[n]=\left\|\tilde{\text{s}}[n]\right\|=\sqrt{{\left(\tilde{\text{s}}[n]\right)}^{T}(\tilde{\text{s}}[n])}$.

Finally, because the zero frequency component (DC), which is present in the signals, can mask information related to the different motion modes experienced by the user and reduce the effectiveness of the signal frequency analysis, it has been removed from the signal of interest:

(3) $${\tilde{s}}_{{\text{rms}}_{0}}[n]={\tilde{s}}_{\text{rms}}[n]-\frac{1}{L}\sum_{l=0}^{L-1}{\tilde{s}}_{\text{rms}}[n-l]$$

where the second term is the signal's mean evaluated using a moving average filter. The selection of the window's length (*L*) is described later.

## 3. Gait Analysis for Step Length Estimation Using Handheld Devices

Before evaluating the step length, step events must be identified. When the sensor is foot mounted, step events can be detected using periods of zero velocity or zero angular rate corresponding to the stance phase, *i.e.*, the period during which the foot is flat on the ground [12]. With handheld devices, these periods are not present. In addition, when the sensor is not body fixed, signal patterns can suddenly vary following a change of the carrying mode or the user's motion. Therefore knowing the nature of the user's motion and the device's carrying mode is critical for designing adaptive algorithms and improving the robustness. To cope with the complexity of the hand case, the process for estimating the user's travelled distance has been divided in three phases: motion mode recognition, step detection and step length estimation. Motion mode recognition and step event detection are performed applying the approach described in details in [15]. This approach is now summarized in Sections 3.1 to 3.5, in order to provide a complete description of the novel step length estimation procedure based on a handheld device.

### 3.1. Motion Mode Recognition

The recognition of the motions of the subject and his/her hand is considered as a classification problem. Six motion modes that are typical for portable device users have been identified, namely:

1. Static: the subject is not moving even if a slight motion occurs.
2. Walking with a swinging hand: the user is walking holding the portable device in the hand.
3. Walking with a texting hand: the user is walking while typing or reading a message/instruction on the screen's device.
4. Walking with phoning hand: the user is using the device to make or receive a phone call.
5. Walking with the mobile device in the handbag.
6. Irregular motion: this class includes all motions that are not related to a real displacement change of the user.

It has been found that texting, phoning and carrying the device can be considered as a unique class for the purpose to optimize the step detection process [15]. During these three motions, the inertial force sensed by the IMU is primarily due to the lower part of the user's body and the sensor signals present patterns that are close to those recorded with body fixed sensors. Consequently, the number of classes describing the motion modes of interest is reduced to four. The classification process is performed by extracting a set of features from the raw sensor data. These features are computed by dividing the data in windows of 256 samples, corresponding to 2.56 seconds, with a 50% overlap. The window size, indicated as *L* in (3), is selected to be small enough to catch any fast motion's change and large enough to include one complete gait cycle. In addition, a window size *N* = 2*^n^* allows the computation of the Fast Fourier Transform (FFT) used for the analysis in the frequency domain. It is worth mentioning that the proposed FFT analysis is applied on a window that is small enough to target real time applications since the induced lag is around half of the window size, namely 1.28 seconds. Real time functionalities have not yet been implemented. The following features, both in time and frequency domains, are extracted to identify the motion mode: the energies, the variances and dominant frequencies of the IMU signal.

### 3.2. Time Domain Features: Energy Related Features and Signal's Variance

The energy features enable distinguishing between activities of low and high intensity. The energy is evaluated by computing the norm of the accelerometer and the gyroscope's pre-processed measurements, adding and normalizing them over the analysis window as:

(4) $$E_{{\tilde{s}}_{\text{rms}}^{a}}=\frac{1}{N}{\sum_{n=0}^{N-1}{\tilde{s}}_{{\text{rms}}_{0}}^{a}}^{2}\left[n\right]$$

(5) $$E_{{\tilde{s}}_{\text{rms}}^{\omega }}=\frac{1}{N}{\sum_{n=0}^{N-1}{\tilde{s}}_{{\text{rms}}_{0}}^{\omega }}^{2}\left[n\right]$$

where *N* is the length of the analysis window ${\tilde{s}}_{{\text{rms}}_{0}}^{a}[n]$ and ${\tilde{s}}_{{\text{rms}}_{0}}^{\omega }[n]$ are the pre-processed components of the accelerometer and gyroscope signals, respectively. This feature is used to distinguish the static from the dynamic state.

The swinging mode experiences much higher amplitudes of angular rate and acceleration energies than the other states, which are the texting, phoning and bag carrying motion modes. Consequently, the variance of the gyroscope signal is also used to distinguish the above states. To refine the classes' characterization, the variances of the gyroscope and accelerometer signals are also evaluated. This statistical measurement is defined as the average of the squared differences from the mean:

(6) $${\sigma^{2}}_{{\tilde{s}}_{{\text{rms}}_{0}}^{a}}=\frac{1}{N}{\sum_{n=0}^{N-1}\left({\tilde{s}}_{{\text{rms}}_{0}}^{a}[n]-\frac{1}{N}\sum_{n=0}^{N-1}{\tilde{s}}_{{\text{rms}}_{0}}^{a}[n]\right)}^{2}$$

(7) $${\sigma^{2}}_{{\tilde{s}}_{{\text{rms}}_{0}}^{\omega }}=\frac{1}{N}{\sum_{n=0}^{N-1}\left({\tilde{s}}_{{\text{rms}}_{0}}^{\omega }[n]-\frac{1}{N}\sum_{n=0}^{N-1}{\tilde{s}}_{{\text{rms}}_{0}}^{\omega }[n]\right)}^{2}$$

This allows the swinging mode to be identified as the variances of both gyroscope and accelerometer signals are larger than in the other cases. This feature is also used to recognize irregular motions characterized by a sudden increase of the variance for both inertial data but without observing any signal periodicity.

### 3.3. Frequency Domain Features: Dominant Frequencies

**Figure 1.**
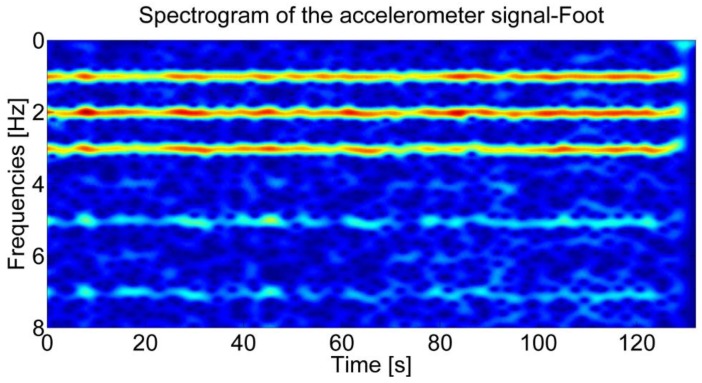
Spectrogram of the accelerometer signal for the sensor mounted on the walking user's foot.

The study of human gait has shown that walking is characterized by a fundamental pattern that is not subjected to inter and intra individual variations and that is produced by the cyclic repetition of the stance and the swinging phases of the foot [17,18]. By analyzing the accelerations in the frequency domain, the human gait periodicity can be captured. Since the signal produced by the walk is not stationary, *i.e.*, the signal's statistical properties change over time, the frequency analysis is performed using the Short Time Fourier Transform (STFT) [19]. This signal processing technique is based on the fragmentation of the input signal in short temporal windows where the signal is assumed to be stationary. The STFT approach, despite its poor time-frequency localization properties, has been selected for its low computational cost and for its non-parametric nature. The spectrogram, a time varying spectral representation of the signal, is then obtained by squaring the absolute value of the STFT. In Figure 1, the spectrogram of the accelerometer signal is reported for a walking subject with the sensor on the foot. Three dominant frequencies, *i.e.*, maximal frequencies in the spectrogram produced by the three main temporal periodicities, are clearly visible.

**Figure 2.**
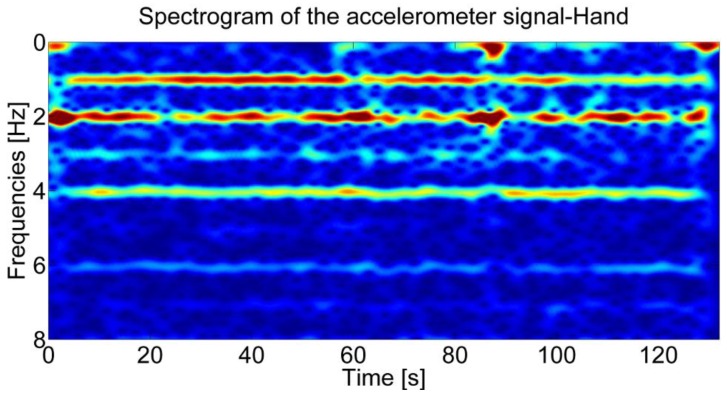
Spectrogram of the accelerometer signal for the sensor placed in the walking user's swinging hand.

As shown in Figure 2, when the sensor is held in hand, three dominant frequencies are also identifiable in the spectrogram of the accelerometer signal for a subject walking alternatively with the sensor in the swinging and in the texting modes. As shown in [15,20], the first two dominant frequencies can be used to distinguish irregular motion and walking mode.

Similar analysis can be conducted with the handheld gyroscope signal to distinguish between different motion modes. Indeed a user walking with the sensor in the swinging hand induces peaks in the spectrogram of the gyroscope signal, due to the periodic rotation of the arm during its swinging phase. Conversely, when the sensor is in the texting mode, phoning hand mode or in the user's bag, dominant peaks are not produced by the gyroscope signal.

### 3.4. Motion Mode Decision Tree

**Figure 3.**
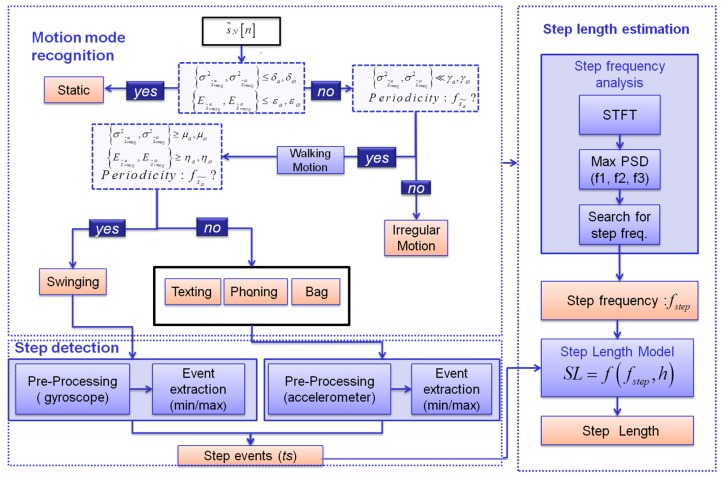
Block diagram of the algorithm for the user's step length estimation using a handheld sensor.

As shown in Figure 3, all above features are integrated in a multivariate decision tree classifier. In Figure 3 the three phases, namely motion mode recognition, step detection and step length estimation necessary to estimate the user's travelled distance, are also represented.

The tree classifier selects one state among all possible motion states represented by the tree leaves. The selection is performed at each node through several tests using the features previously described. The decision tree initially differentiates static and dynamic activity using the energies and variances of MEMS signals. If the activity is classified as dynamic, the periodicity of the accelerations is analyzed. The latter reflects the periodicities of human gait making it possible to distinguish normal walking and irregular motions. Irregular motion is generally characterized by very high values of the variance in a short period of time, e.g., when the user is pulling out a device from a bag without moving. Finally, the features described in Sections 3.2–3.3 enable distinguishing the states of swinging and texting, phoning and carrying the bag. The classifier achieves high performance independently from the considered subject because the selected features are characterized by a high inter-class distance. More details about the classification process can be found in [15].

### 3.5. Step Identification

Once the motion state is determined, an adaptive step detection algorithm is applied. If the IMU is held in a swinging hand, the experienced inertial force results from the gross motion of the subject plus the swinging motion. However biomechanical studies have shown that a synchronization exists between the swinging of the arm and the foot motion during walking. Specifically, it has been observed that during normal walking, the arm swing is produced for decreasing the reaction momentum about the vertical axis of the foot [21]. When the left foot is in the stance phase, a positive torque, produced by the arm swing, allows the advancement of the right leg. This relation, which is valid in the absence of particular pathologies, allows the identification of step events from the swing of the user's arm. The step information is extracted by using angular rates because the periodic rotation of the arm produces a sinusoidal pattern in the gyroscopes' signals. Subsequently, peaks in the gyroscopes' signals are determined and the up and forward hand's motions are detected along with the synchronized swing phase of the foot [15].

When the sensor is placed in the user's texting/phoning hand or carried in the bag/trouser pocket, it is mainly reflecting the general motion of the user. Even in these cases, periods of zero velocity cannot be determined in the accelerometer signal. However the repetition of swing phases produces negative and positive peaks in the accelerometer signal pattern. These peaks are used to mark step occurrences. Signal peak detection is performed by recognizing a local maximum or minimum within the sliding window and by exploiting algorithms based on adaptive threshold. Thanks to the adaptive feature, the peak detection algorithm is independent from the level of the signal energy and therefore the variations that the hand could undergo. To minimize the probability of false peak detection, a dedicated preprocessing phase of IMU signals has been added. Inertial signals are low-pass filtered using a 10th order Butterworth filter with a 3-Hz cut-off frequency, which produces an undistorted signal for detecting the fundamental frequency produced by the step events. The cut-off frequency value is selected considering the typical range of step frequencies experienced by a pedestrian walking at a normal speed [22]. Finally the algorithm adopts the mean value over a sliding window as the threshold for detecting peaks. If a sample in the window gives a larger value than the adaptive threshold, a peak is identified and a step is detected.

## 4. Step Length Model

Once a step is detected, the next stage for tracking pedestrians is to determine their position using the estimated step length. With body fixed sensors, it has been shown in literature that a linear relationship between step length and step frequency exists [9,22]. This can be intuitively understood and expanded to handheld devices. If a pedestrian walks faster, both step's length and step frequency will increase. Biomechanical studies have shown that in general, the user's step length is proportional to the length of the user's leg and subsequently to the user's height [17]. Starting from these results, a new step length model for handheld devices has been designed and empirically validated. It combines the step frequency and user's height.

The best linear relationship between frequency and step length was found to be the one weighted by the user's height and is given by:

(8) $$\begin{array}{l} s=h\cdot (a\cdot f_{\text{step}}+b)+c\\ K=\{a,b,c\}\in \mathbb{R} \end{array}$$

where *h* is the user's height, *f_st_*_ep_ is the step frequency and *K* is a set of parameters. A universal and a calibrated model are proposed. The universal model has been developed for giving a first approximation for any filter that would offer tuning functionalities. As detailed in Section 5 the universal model is based on a set of constants trained using 12 test subjects while the calibrated model tailors the set of constants for each subject.

Recursive Least-Squares (RLS) [23] is used to determine the calibrated set of model's parameters. The approach is based on the recursive evaluation of the optimum parameters by minimizing the sum of squared residuals between the true step length and the predicted step length. The universal set of parameters *K* is used for the initial solution **x**_0_:

(9) $${\hat{\text{x}}}_{k+1}={\hat{\text{x}}}_{k}+{\left({\text{H}}^{T}\text{H}\right)}^{-1}{\text{H}}^{T}(\text{s}-\text{Hx})$$

with:

(10) $$\begin{array}{c} {\text{x}}_{0}={{\left[\begin{array}{ccc} a & b & c \end{array}\right]}^{T}}_{\text{universal}},\\ \text{H}=\left[\begin{array}{ccc} h_{{\text{user}}_{1}}f_{{\text{step}}_{1},{\text{user}}_{1}} & h_{{\text{user}}_{1}} & 1\\ \vdots & \vdots & \vdots \\ h_{{\text{user}}_{n}}f_{{\text{step}}_{k},{\text{user}}_{n}} & h_{{\text{user}}_{n}} & 1 \end{array}\right],\\ \text{s}={\left[\begin{array}{ccc} s_{{\text{user}}_{1},1} & \cdots & s_{{\text{user}}_{n},k} \end{array}\right]}^{T}. \end{array}$$

**s** comprises the true step lengths for all epochs between 1 and k. **H** comprises the step frequencies and users' heights for n test subjects over k epochs. The set of fitted parameters is determined when the convergence criteria over **x̂***_k_*_+1_ − **x̂***_k_* is achieved. True step lengths are evaluated following the procedure described in Section 5.1 while predicted step lengths are obtained using the model in Equation (8).

### 4.1. Step Frequency Evaluation

The proposed step length model strongly relies on the extraction of the user's step frequency and its quality. In most of the works, the step frequency is calculated in the time domain by detecting steps and computing the inverse of their duration [8]. In order to render the algorithms more robust, the step's cadence estimation proposed herein was designed independently from the step detection process. Indeed it is directly evaluated in the frequency domain by computing the FFT of handheld accelerometer signals. This is a critical observation as it ensures that the algorithms constituting the step length estimation process remain uncorrelated.

Since the sensor is located in the hand, the step's frequency cannot be directly extracted from the hand signals. In order to tailor the model to the handheld case, a dedicated analysis was conducted to accurately relate the handheld signal frequencies to the walking cycle. Work concentrated on the coordination between legs, arms and hands.

**Figure 4.**
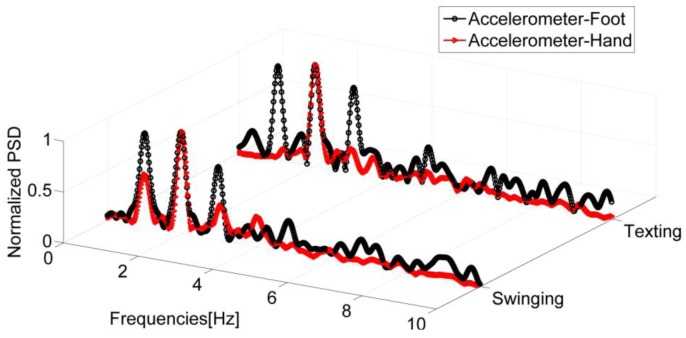
Normalized PSD of the accelerations sensed by the foot mounted sensor and the one in the swinging and texting hand. For both motion modes, the strongest frequency is coupled with the step event.

**Figure 5.**
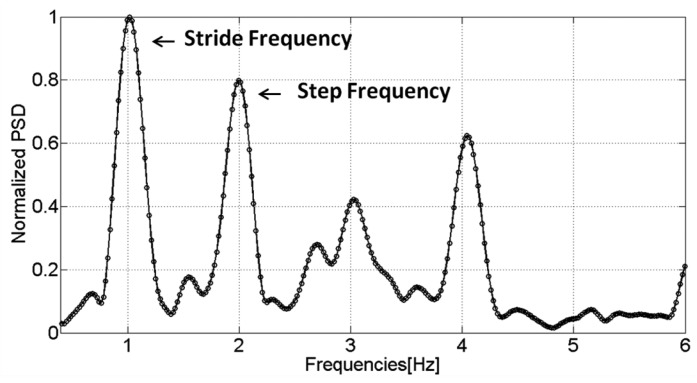
Normalized PSD for the accelerometer sensed by the user's swinging hand. Here, the strongest frequency is coupled with stride events.

The examined signals are those of two IMUs, the first one placed in the hand and the second, rigidly fixed on the foot. In Figure 4, the Power Spectral Densities (PSDs) are shown for the accelerometers of both IMUs in the swinging and texting cases. It is observed that the dominant frequency peaks are centered on the same values for both sensors, which shows that the step frequency can be derived even if the sensor is not located on the foot or is not body fixed. Furthermore, the strongest frequency of the accelerometer signal, *i.e.*, the frequency with the maximum power, is not always coupled with the same event of the walking gait cycle. Sometimes it is coupled with the step event (Figure 4) and sometimes, especially for faster speeds, with the stride event. This is further illustrated in Figure 5 where the PSD extracted from the signal of a sensor in the user's swinging hand is reported. The PSD is estimated using the Welch periodogram technique [24]. It is performed by dividing the signal into overlapping blocks and averaging the squared magnitude FFTs (Fast Fourier Transform) of the signal blocks. When compared with the use of the standard periodogram, the main advantage of this method is to minimize the variance, which renders this spectrum estimator unbiased. The spectrum analysis shows that the faster the speed is, the greater the chances are that the strongest frequency and stride event are coupled.

Consequently a binary classifier was designed to identify step frequencies from the extracted hand frequencies. After selecting the strongest frequency, the classifier applies the following decision rules:

(11) $$\{\begin{array}{cc} \text{if} & f_{\text{strongest}}>\tau \Rightarrow f_{\text{strongest}}=f_{\text{step}}\\ \text{if} & f_{\text{strongest}}<\tau \Rightarrow f_{\text{strongest}}=f_{\text{stride}} \end{array}$$

**Figure 6.**
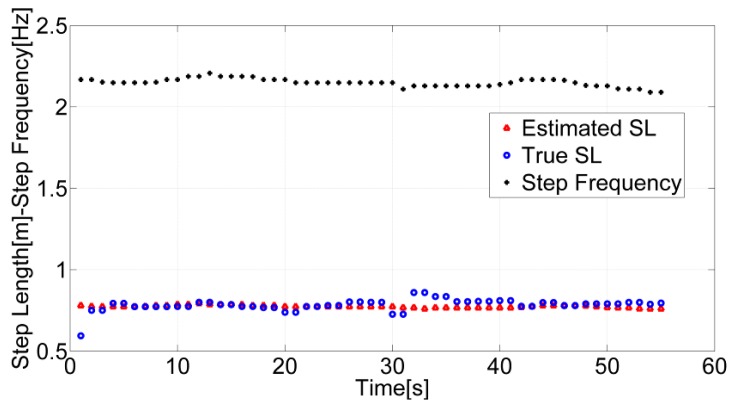
Estimated, true step lengths and step frequencies computed with signals from a handheld IMU when the user is walking with his hand swinging.

A 1.4 Hz threshold *τ* has been selected based on the fact that for the normal walking case the range of frequencies for a human step is generally above 1.6 Hz. This assumption has been validated experimentally by analyzing the steps' length of the test subjects. However, further analysis should be performed to investigate how the users' age affects step frequency. In the proposed algorithm, if the detected frequency is the stride frequency, the step frequency can be derived by multiplying the strongest frequency value by two. The step frequencies, extracted after applying the binary classifier, true step lengths and the estimated ones by using the step length model and the universal set of parameters are shown in Figure 6 for a user walking with the sensor located in the swinging hand.

## 5. Experimental Assessment

### 5.1. Data Collections

**Figure 7.**
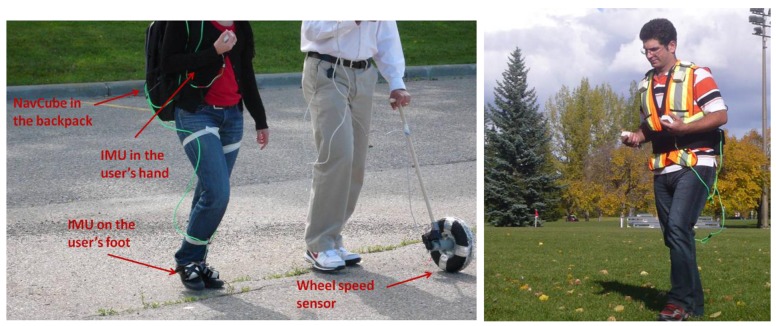
(**Left side**) Data collection set up for training the parameters of the step model. The subject walks at different speeds with one IMU in the hand and one on the foot. A second person paces the test subject by using a wheel speed sensor; (**Right side**) Data collection set up for assessing the step length model. The subject walks at his preferred speed with two (only one is used for data analysis) IMUs in the hands and one on the foot. The NavCube is carried at the waist.

**Figure 8.**
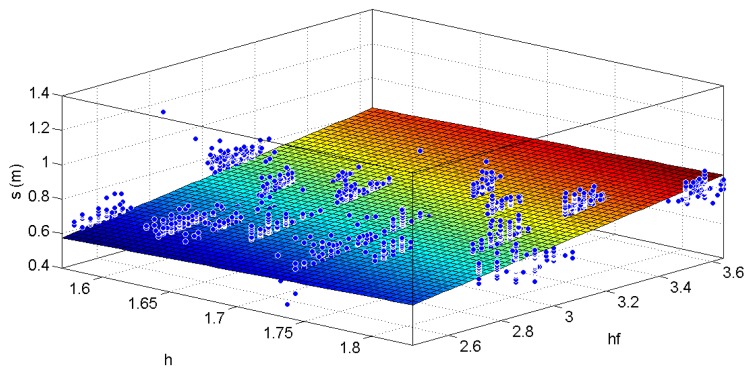
Linear fitting of the true step lengths (blue dots) with the user's heights (h) and the product of the strongest dominant frequencies with the user's heights (hf) at different walking speeds and hand's motions. The outcome is the universal set *K* in the step length model Equation (8).

In order to train and assess the performance of the proposed step length model, several field tests were performed. The first type of data collections has been performed to fit the three constants characterizing the set of parameters, indicated as *K* in Equation (8), defining the universal model. The above parameters have been computed using data collected by 12 different subjects: six men and six women between twenty-forty years old. They walked along a 200 m straight line on a parking lot, at three different speeds, for a total of approximately 600 m. True step lengths were measured using a wheel speed sensor whose records were time tagged with GPS time and inertial signals from a foot mounted IMU. As shown in the left side of Figure 7, the wheel speed sensor was controlled by an instructor, whose role was to pace the pedestrian. The test subjects were requested to walk at slow speed (about 0.8 km/h), intermediate speed (about 1.8 km/h) and fast speed (about 4 km/h) with the hand in texting and swinging motion modes. Foot mounted inertial signals were also used to assess the proposed step detection algorithms with a handheld IMU. Detection of the foot stance phases was performed by assessing the acceleration variances. Universal parameters are the outcome of fitting Equation (8) to all 12 datasets simultaneously. Figure 8 shows the best fitting of the step length model as a function of the user's height and the product of the user's height with the strongest gait frequency extracted from the handheld device. The true step lengths are depicted in blue. The higher the latter product is the faster the pedestrian walking speed is.

For assessing the performance of the proposed model in the position domain, a second experiment was conducted in an open soccer field with different test subjects than the ones who participated to the fitting of the universal step length model. All data collections were performed using a multi-sensor navigation platform, the NavCube [25], developed at the University of Calgary. The platform includes a Novatel receiver and supports up to ten 6DoF Analog Devices ADIS16375 IMUs. All data were synchronized with GPS time. During these field tests, two IMUs were used: one placed in the hand and one mounted on the user's foot, as shown in the right side of Figure 7. The foot mounted IMU served as a reference for the assessment of the step detection process. Five women and five men were equipped with this hardware setup and requested to walk twice along a curved route of about 300 m, for a total of 600 m. During the first run, the subjects were holding the inertial sensor in a swinging hand and for the second run, they were asked to change to the texting mode without stopping their walk. This corresponds to a natural change of the sensor carrying mode without stopping the walk. Because the pedestrian's walk is different for each person, the set of parameters *K* can be optimized individually through a calibration phase. To assess the benefit of calibrating the model, each subject was also requested to perform two straight paths in texting and swinging modes. This part of the test was used to find a calibrated set of *K* for each subject. For this purpose, “true” step lengths were evaluated by interpolating post-processed differential GPS positions over each identified step. Finally iterative least-squares (LS) process was applied to determine the calibrated set K_n_, with a convergence achieved at the n^th^ iteration.

### 5.2. Experimental Results

In order to assess the performance of the proposed step length model, the user's motion mode and step events are first identified. The performances of the motion mode classifier, which are evaluated for the soccer field tests, are reported in Table 1. The rows of the table show the tested activities, namely swinging and texting modes, while the columns indicate the predicted motion modes. The confusion matrix reports along its principal matrix the percentages of correct detection for each state while the off diagonals report the percentages of misclassification. In Table 2 the probability of correct detection of the user's motion mode is also reported for each test subject along with the percentage of correct step detection. However an extensive validation of the classifier and of the step detection algorithms has been proposed in [15].

Then, the percentage of error over the travelled distance has been computed for each test subject with:

(12) $$\varepsilon =\frac{{\text{d}}_{\text{handheld}}}{{\text{d}}_{\text{GPS}}}$$

where d_handheld_ is the total traveled distance estimated using the step length model and d_GPS_ is the reference distance evaluated using post-processed GPS carrier phase signals in a differential mode. The accuracy of the post-processed solution with a 1 km baseline in the open sky was better than 1 cm. A pedestrian dead reckoning approach has been used to compute the total traveled distance. It is based on the sum of each step displacement vector p_t_, estimated at the time t and starting from the initial position p_0_ as:

(13) $$\begin{array}{l} {\text{p}}_{t}={\text{p}}_{t-1}+s_{t}{\left[\begin{array}{cc} \cos\theta_{t} & \sin\theta_{t} \end{array}\right]}^{T}\\ {\text{d}}_{\text{handheld}}=\sum_{t=0}^{\text{end}-1}\left\|{\text{p}}_{t}-{\text{p}}_{t-1}\right\| \end{array}$$

where *s_t_* is the step length evaluated by applying the proposed model and *θ_t_* is the GPS-based walking direction over one step. This angle has been extracted from the GPS trajectory, post-processed in differential mode, for translating the estimated displacement information into the positioning domain. As seen mathematically in Equations (12) and (13), using these post-processed headings does not affect the estimation of the distance travelled error. The error percentages are reported in Table 1 for all test subjects using both the universal and the calibrated set of parameters defined in Equation (8). Men are indicated with “M” and women with “W”. The number of iterations necessary to achieve the LS convergence in the calibration process is also reported.

Results show that even for the universal model, most of the travelled distance errors are between 4 and 6%. Two larger percentages are obtained for the male subjects M4 and M5 with 8 and 9% respectively for the universal model. However, with the calibrated handheld based step length model, the results significantly improve and the highest percentage becomes 5%.

**Figure 9.**
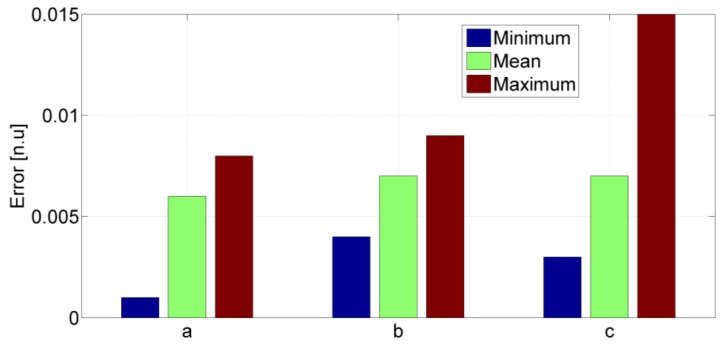
Minimum, mean and maximum absolute differences between “fitted” and “universal” parameters of the proposed step length model.

Overall, the performance of the handheld based step length model is comparable to those of models proposed for body fixed sensors [5,6]. The quality of the universal model is further confirmed by the low number of iterations required to reach convergence. In order to further assess the validity of the universal model, the absolute differences between fitted and universal parameters have been evaluated for each test subject. In Figure 9, minimum, maximum and mean values of the above differences show the variations between fitted and universal parameters.

**Figure 10.**
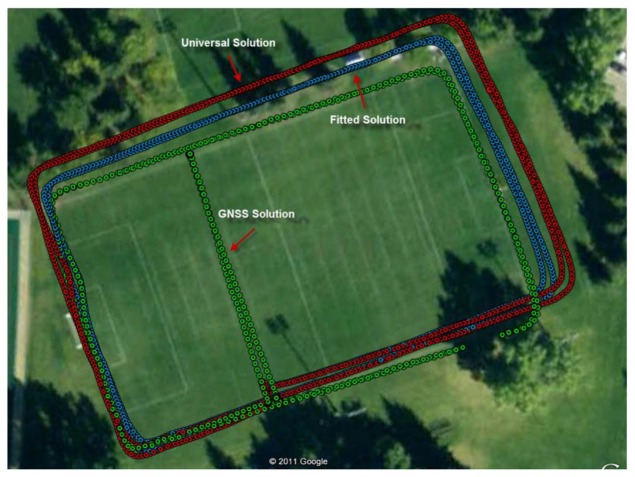
Reference path in green and estimated trajectories: modeled step length with the universal parameters in red and with the calibrated parameters in blue for the test subject with the worst performance (M5).

Finally, Figure 10 shows the three walking paths for the worst results (M5). It can be observed that the universal model overestimates the travelled distance but that the calibrated model improves the overall performance.

It is worth mentioning that the test subject M5 was significantly taller than most of the subjects that participated in the training phase. This explains the larger disagreement between the model and the truth. Increasing the number and the variety of training subjects would improve the performance of the universal model.

## 6. Conclusions

In this paper, a step length model for evaluating the distance travelled by a pedestrian holding an IMU in a hand has been proposed. Before computing the distance walked over a step, the carrying mode of the device, *i.e.*, texting, phoning or arm swing, and the user motion are identified. By using adaptive algorithms, step events are detected from the signals recorded in the hand and for all arm/hand motions. The proposed step length model for smart phone users combines the user's step frequency, the user's height and a set of three variables. The algorithms developed for estimating the pedestrian's step frequency using non-body fixed sensors have been presented. The fact that the strongest frequency of the signal extracted from the handheld IMU can be coupled either with the step or the stride of the pedestrian is used to estimate the step cadence. Performance of the model was assessed in the position domain with a universal set of parameters and one fitted for each person. The experimental tests have demonstrated percentages of error over the travelled distance between 2.5% and 5%. The latter are similar to those achieved in the literature but with sensors rigidly attached to the body. These results are applicable to autonomous navigation and tracking of pedestrians using smart phones. In addition, the proposed algorithms are processing the IMU signals in windows between 1.5 to 2.5 seconds, which enables real time implementation. Further improvements of the step model could be obtained by increasing the typology of subjects involved in the model's training phase.

## Figures and Tables

**Table 1.** Confusion Matrix for the motion mode classifier.

**Test Motions**	**Classified as:**		
	**Texting**	**Swinging**	**Irregular**
**Texting**	**100%**	**0%**	**0%**
**Swinging**	**0%**	**98%**	**2%**

**Table 2.** Metrics for Evaluating the Handheld Based Step Length Model.

**Subject**	**%P_det_** **(motion)**	**%P_det_** **(steps)**	**%DistanceTravelled**		**Convergence Iterations**
			**Universal Model**	**Fitted Model**	
**M1**	**100**	**99**	**5.8**	**5**	**4**
**M2**	**100**	**100**	**4.8**	**4.3**	**3**
**M3**	**99**	**100**	**5**	**4.5**	**3**
**M4**	**100**	**99**	**8**	**4.2**	**6**
**M5**	**100**	**100**	**9**	**3.8**	**7**
**W1**	**98**	**97**	**5.2**	**4.3**	**4**
**W2**	**100**	**100**	**3.2**	**2.5**	**3**
**W3**	**98**	**99**	**4.5**	**4**	**3**
**W4**	**100**	**98**	**5.6**	**5**	**3**
**W5**	**100**	**100**	**5.8**	**5**	**4**
**Mean**	**99.6**	**99.2**	**5.7**	**4.2**	**4**
